# Supplementary material for: High Virulence and Multidrug Resistance of Escherichia coli Isolated in Periodontal Disease
Source: Microorganisms. 2022 Dec 23;11(1):45. doi: 10.3390/microorganisms11010045 (PMC9863014; doi:10.3390/microorganisms11010045)
Supplement: Supplementary file 1 [file microorganisms-11-00045-s001.zip › Supplementary material S1.pdf]

Supplementary material S1.

Primers used to amplify the virulence genes of *E. coli.*

The PCR method and the sequences of the primers to identify the virulence genes were described by:

Momtaz, H; Karimian, A; Madan, M; Dehkordi, S.F; Ranjbar, R; Sarshar, M; Souod, N. Uropathogenic *Escherichia coli* in Iran: Serogroup distributions, virulence factors and antimicrobial resistance properties. *Ann. Clin. Microbiol. Antimicrob.* **2013**, 12:8.

Rodriguez-Siek KE, Giddings CW, Doetkott C, Johnson TJ, Fakhr MK, Nolan LK. Comparison of Escherichia coli isolates implicated in human urinary tract infection and avian colibacillos. *Microbiol*. **2005**, *151,* 2097-2110.

**Gene**	**Name**	**Sequence 5'-3'**	**PCR product size (bp)**
**ADHESINS**			
papA	PapA-F	ATGGCAGTGGTGTCTTTTGGTG	717
	PapA-R	CGTCCCACCATACGTGCTCTTC	
papGI	papGJ96-F	TCGTGCTGAGGTCCGGAATTT	461
	papGJ96-R	TGGCATCCCCCAACATTATCG	
papGII	papGIA2-F	GGGATGAGCGGGCCTTTGAT	190
	papGIA2-R	CGGGCCCCCAAGTAACTCG	
papGIII	prsJ96-F	GGCCTGCAATGGATTTACCTGG	258
	prsJ96-R	CCACCAAATGACCATGCCAGAC	
fimH	FimH-F	TGCAGAACGGATAAGCCGTGG	508
	FimH-R	GCAGTCACCTGCCCTCCGGTA	
afa	Afa-F	GCTGGGCAGCAAACTGATAACTCTC	750
	Afa-R	CATCAAGCTGTTTGTTCGTCCGCCG	
sfaS	SfaS-F	GTGGATACGACGATTACTGTG	244
	SfaS-R	CCGCCAGCATTCCCTGTATTC	
iha	IHA-F	CTGGCGGAGGCTCTGAGATCA	827
	IHA-R	TCCTTAAGCTCCCGCGGCTGA	
focG	FocG-F	CAGCACAGGCAGTGGATACGA	364
	FocG-R	GAATGTCGCCTGCCCATTGCT	
bmaE	bmaE-F	ATGGCGCTAACTTGCCATGCTG	507

	bmaE-R	AGGGGGACATATAGCCCCCTTC	
**TOXINS**			
cnf1	cnf-F	AAGATGGAGTTTCCTATGCAGGAG	498
	cnf-R	TGGAGTTTCCTATGCAGGAG	
hlyA	hly-F	AACAAGGATAAGCACTGTTCTGGCT	1177
	hly-R	ACCATATAAGCGGTCATTCCCGTCA	
tsh	Tsh-F	ACTATTCTCTGCAGGAAGTC	824
	Tsh-R	CTTCCGATGTTCTGAACGT	
usp	usp-F	ACATTCACGGCAAGCCTCAG	440
	usp-R	AGCGAGTTCCTGGTGAAAGC	
set-1	set-1-F	GTGAACCTGCTGCCGATATC	147
	set-1-R	ATTTGTGGATAAAAATGACG	
astA	astA-F	ATGCCATCAACACAGTATAT	110
	astA-R	GCGAGTGACGGCTTTGTAGT	
**IRON UPTAKE SYSTEMS**			
iuc	Iuc-F	ATGAGAATCATTATTGACATAATTG	1482
	Iuc-R	CTCACGGGTGAAAATATTTT	
iroN	IRONEC-F	AAGTCAAAGCAGGGGTTGCCCG	665
	IRONEC-R	GACGCCGACATTAAGACGCAG	
irp2	Irp2-F	AAGGATTCGCTGTTACCGGAC	413
	Irp2-R	AACTCCTGATACAGGTGGC	
feoB	FEOB-F	AATTGGCGTGCATGAAGATAACTG	470
	FEOB-R	AGCTGGCGACCTGATAGAACAATG	
fyuA	FyuA-F	TGATTAACCCCGCGACGGGAA	787
	FyuA-R	CGCAGTAGGCACGATGTTGTA	
ireA	IREA-F	GATGACTCAGCCACGGGTAA	254
	IREA-R	CCAGGACTCACCTCACGAAT	
**PROTECTINS**			

kpsMT	kpsII-F	GCGCATTTGCTGATACTGTTG	272
	kpsII-R	CATCCAGACGATAAGCATGAGCA	
ompT	ompT-F	ATCTAGCCGAAGAAGGAGGC	559
	ompT-R	CCCGGGTCATAGTGTTCATC	
iss	Iss-F	ATCACATAGGATTCTGCCG	309
	Iss-R	CAGCGGAGTATAGATGCCA	
traT	TraT-F	GGTGTGGTGCGATGAGCACAG	290
	TraT-R	CACGGTTCAGCCATCCCTGAG	
**PATHOGENIC ISLANDS**			
malX	MALX-F	GGACATCCTGTTACAGCGCGCA	925
	MALX-R	TCGCCACCAATCACAGCCGAAC	

Primers used for detection of phylogroups in *E. coli.*

The PCR method and the sequences of the primers to identify the virulence genes were described by:

Clermont, O.; Christenson, JK.; Denamur, E.; Gordon, DM. The Clermont *Escherichia coli* phylo-typing method revisited: improvement of specificity and detection of new phylo-groups. *Environ. Microbiol. Rep.* **2013**, *5,* 58-65.

PCR reaction	Target	Sequence (5´-3´)	PCR product size (bp)
Multiplex	*chuA*	(F) ATGGTACCGGACGAACCAAC<br>(R) TGCCGCCAGTACCAAAGACA	288
	yjaA	(F) CAAACGTGAAGTGTCAGGAG<br>(R) AATGCGTTCCTCAACCTGTG	211
	TspE4.C2	(F) CACTATTCGTAAGGTCATCC<br>(R) AGTTTATCGCTGCGGGTCGC	152
	arpA	(F) AACGCTATTCGCCAGCTTGC<br>(R) TCTCCCCATACCGTACGCTA	400
Group E	*arpA*	(F) GATTCCATCTTGTCAAAATATGCC<br>(R) GAAAAGAAAAAGAATTCCCAAGAG	301
Group C	*trpA*	(F) AGTTTTATGCCCAGTGCGAG	219

		(R) TCTGCGCCGGTCACGCCC	
Internal control	*trpA*	(F) CGGCGATAAAGACATCTTCAC<br><br>(R) GCAACGCGGCCTGGCGGAAG	489
